# A systematic review of omics discovery studies to identify pertinent metabolic pathways for locally advanced rectal cancer in response to neoadjuvant chemoradiotherapy

**DOI:** 10.1007/s11306-025-02319-y

**Published:** 2025-08-21

**Authors:** Aaron Kler, Matthew Fok, Gabrielle J. Grundy, Marco Sciacovelli, Warwick B. Dunn, Dale Vimalachandran

**Affiliations:** 1 Institute of Systems Biology, Molecular and Integrative Biology, University of Liverpool, Liverpool, UK; 2 Department of Colorectal Surgery, Countess of Chester Foundation Trust, Chester, UK; 3 Department of Biochemistry, Cell and Systems Biology, Centre for Metabolomics Research, Institute of Systems, Molecular, and Integrative Biology, University of Liverpool, Liverpool, L69 7ZB UK

**Keywords:** Metabolic, Metabolomics, Neoadjuvant, Rectal cancer

## Abstract

**Background:**

Locally advanced rectal cancer (LARC) has variable responses to neoadjuvant therapy (NAT). Therefore, identifying changes in biological pathways involved when LARC is treated with NAT is crucial for developing treatments to improve clinical outcomes, as NAT is both variable and unpredictable. Although individual studies have attempted to discern how the response differs at a transcriptomic, proteomic and metabolomic level, there has not been a unifying systematic review discerning the key changes in metabolic pathways in this patient population.

**Aim of review:**

This systematic review aims to understand how metabolomics, proteomics and transcriptomics can demonstrate how the perturbed metabolic pathways of the NAT response in LARC can provide targets for further clinical research.

**Key scientific concepts of review:**

Thirteen studies met the inclusion criteria, including seven metabolomic, five proteomic, and one transcriptomic study. Metabolomic analyses revealed consistent alterations in amino acid metabolism, the tricarboxylic acid (TCA) cycle, and glycerophospholipid metabolism. Proteomic findings supported these results, highlighting disruptions in glycolysis and gluconeogenesis. Joint pathway analysis demonstrated a strong correlation (*r* = 0.99, *p* < 0.0001) between metabolic changes observed across omics platforms. Key pathways such as alanine, branched-chain amino acid, and aspartate metabolism were commonly altered and may contribute to radio-resistance through enhanced energy production, reactive oxygen species (ROS) neutralization, and DNA repair mechanisms. The convergence of multi-omic data underscores the biological relevance of these metabolic reprogramming events. However, due to the limited availability of transcriptomic data meeting inclusion criteria, these findings are primarily driven by metabolomic and proteomic analyses, which constrains the extent of full multi-omic integration. Future studies should aim to validate these findings in clinical cohorts and explore how targeting these “survival” pathways could optimize treatment response in LARC.

**Supplementary Information:**

The online version contains supplementary material available at 10.1007/s11306-025-02319-y.

## Introduction

Colorectal cancer (CRC) is a condition characterized by abnormal cell growth specifically within the colon or rectum and is the fourth most common cancer worldwide (Mármol et al., 2017). Cancers of the rectum (defined as tumours within 15 cm of the anal verge) and rectosigmoid junction account for 30% of all CRC diagnosed. Although surgical resection is the first-line treatment for locally advanced colon cancers, locally advanced rectal cancers (LARC) are typically treated with neoadjuvant therapy (NAT) prior to surgical resection. These include short-course radiotherapy (SCRT), which delivers 25 Gy in five daily fractions over one week followed by delayed surgery (Kapiteijn et al., 2001; Swedish Rectal Cancer Group, 1997), and long-course chemoradiotherapy (LCRT), which combines approximately 50.4 Gy in 28 fractions over five to six weeks with concurrent fluoropyrimidine-based chemotherapy (Roh et al., 2009; Sauer et al., 2004). More recently, total neoadjuvant therapy (TNT) has emerged, integrating both the full dose of systemic adjuvant chemotherapy, such as FOLFOX or CAPOX, and radiation prior to surgery, with the aim of treating micrometastatic disease earlier and increasing rates of pathological complete response (pCR) (Cercek et al., 2018).

While these approaches have successfully reduced local recurrence rates, they have not yet demonstrated consistent improvements in overall survival. Treatment response to NAT is both varied and unpredictable. A pathological complete response (pCR) to NAT is estimated at 15–20% (Burbach et al., 2015), whilst 40–45% show evidence of downstaging; however, 20–30% have no response at all (Kaminsky-Forrett et al., 1998; Kuremsky et al., 2009; Onaitis et al., 2001). Therefore, there is a requirement to investigate the biological changes associated with NAT to understand how and why patients respond differently, to guide treatment.

Variability in NAT response is multifactorial. To aid our understanding, it is important to appreciate the metabolic reprogramming changes in CRC and related oncogenic drivers as metabolic reprogramming has been associated with resistance to NAT or worst response/prognosis. CRC can be subclassified according to consensus molecular subtyping, with CMS3 subtype (Guinney et al., 2015) (metabolic) characterised by deep metabolic regulation. This leads to different metabolic phenotypes that characterise cancer activity (Guinney et al., 2015), such as the variability in the response to NAT.

A typical metabolic reprogramming hallmark of CRC includes changes in glucose metabolism. In human cells, glucose is oxidised to pyruvate through glycolysis and pyruvate then enters the tricarboxylic acid (TCA) cycle and subsequent oxidative phosphorylation pathways. However, in CRC and many other cancers, cells undergo metabolic reprogramming to favour aerobic glycolysis, known as the Warburg effect, while still maintaining oxidative phosphorylation. The balance between aerobic glycolysis and oxidative phosphorylation varies depending on the specific tumour type and stage. There are multiple reasons for preferential aerobic glycolysis and one suggested theory is to satisfy the need for additional biomacromolecules (Ly et al., 2020) as glycolysis can shunt biosynthetic precursors into anabolic reactions that branch from this pathway, contributing to production of nucleosides, lipids, and/or proteins for rapidly proliferating cells.

Metabolic reprogramming, which enhances survival against radiation, is also a common trait of cancer cells. Radioresistant cancers can reroute metabolism to boost NADPH levels, which enables the reduction of oxidised glutathione. Subsequently, this reduces concentrations of reactive oxygen species (ROS), a typical by-product of radiotherapy/radiation treatment that causes harmful double-stranded DNA (dsDNA) breaks (Cannan & Pederson, 2016). Consequently, the surplus NADPH produced from metabolic rewiring can offer a valuable survival advantage during radiotherapy to prevent dsDNA breaks and increased oxidative stress.

In addition to altered glucose metabolism, metabolism of amino acids such as glutamine can reprogramme to enhance CRC cell survival against radiotherapy and aid proliferation. Cancer cells have demonstrated a dependence on external glutamine despite its status as a non-essential amino acid (NEAA) (Jiang et al., 2016). Glutamine’s functions overlap into glucose metabolism as a carbon source to reload the TCA cycle (Cluntun et al., 2017). Furthermore, glutamine can act as a precursor for lipid or nucleotide synthesis (Pavlova & Thompson, 2016) and de novo synthesis of other NEAAs (Pavlova et al., 2022) to supplement the need for additional biomacromolecules.

Given the metabolic reprogramming changes occurring in CRC, and the unknown changes occurring in LARC in response to NAT, this review aims to assess the literature for metabolomic, proteomic, and transcriptomic studies on patients receiving NAT for LARC to identify the mechanisms of reprogramming metabolic pathways in response to NAT.

## Methods

A systematic literature search was performed following consultation of the Preferred Reporting Items for Systematic reviews and Meta-Analyses (PRISMA) (Shamseer et al., 2015) guidelines according to a predefined protocol. Written consent was not requested as it was not required for this paper. The review was registered in the International Prospective Register of Systematic Reviews (PROSPERO) (ID: CRD42024490866).

### Eligibility criteria

Discovery studies investigating the metabolomic, proteomic and transcriptomic profiles of patients receiving NAT for LARC were included using either blood or tumour samples. Transcriptomic studies using coding mRNA only were included whilst studies examining microRNA (miRNA) and long non-coding RNA (lncRNA) were excluded. Coding mRNA has more phenotypical relevance on a multi-omic stage due to its inherent relationship to proteins, and metabolites. Studies with only one timepoint, paediatric studies, non-English language reports, literature reviews, consensus documents were also excluded.

### Search strategy

A systematic search was performed on 18/06/2025 on Pubmed, Web of Science and Scopus and the Gene Expression Omnibus (GEO) was also utilised to search for transcriptomic data. Two authors (AK and MF) performed the search independently of each other. The search strategy used for all databases was: (((metabolomics OR metabolism OR metabolome OR proteomics OR protein OR proteome OR transcriptomic OR transcriptome OR mRNA OR RNA) AND (colorectal OR colon OR rectal OR rectum OR CRC)) AND (cancer OR tumour OR malignancy)) AND (neoadjuvant OR chemotherapy OR radiotherapy OR chemoradiotherapy OR NCT OR NAT) AND (locally advanced OR locally-advanced).

### Data extraction

Data extraction was performed using a standardised proforma. Data collection included study data such as authorship, country of origin of the corresponding author, publication year, study design, study size and journal of publication. Further data collected included analytical platform used, metabolites, proteins or gene names found to be statistically significant by each independent study, chemoradiotherapy regimen, specimens collected and number of timepoints used for specimen collection.

### Study selection

Studies meeting our review’s eligibility criteria were selected, and their full texts were reviewed. Any discrepancies in eligibility were resolved by discussion between the authors; if there was any unresolved discrepancy, a third author was consulted.

### Statistical analysis

Metabolites, proteins and gene symbols found to be statistically significant to the NAT response as reported in each study were combined between eligible reports. To align with a more refined inclusion criterion for metabolites, only those reported as significantly altered (raw p-value < 0.05) in at least two independent studies were included, and pathway analysis was subsequently performed using MetaboAnalyst 6.0(Pang et al., 2024) to determine the most relevant pathways associated with metabolic changes between NAT-sensitive and NAT-resistant patients in a metabolite-only pathway analysis. Patients categorised as NAT-sensitive/NAT-resistant patients were done so at the classification system used by the included article. Hypergeometric test was applied. No fold changes were incorporated into the analysis. Independent proteomic pathway analysis was used to perform pathway analysis for proteomic studies using QIAGEN Ingenuity Pathway Analysis (IPA). Proteomic pathway analysis was performed using core metabolomics analysis. Transcriptomic data was not utilised due to a lack of available data. Joint pathway analysis was performed using MetaboAnalyst 6.0(Pang et al., 2024) applying Hypergeometric test set to “metabolic pathways only”. Statistical analyses were performed using GraphPad Prism 9.0.

### Methodological quality

The QUADOMICS tool was employed to assess the methodological quality of the studies. QUADOMICs is an adaptation of QUADAS, a tool used in systematic reviews to assess quality specific to omics studies (Lumbreras et al., 2008). Studies scoring ≥ 12/16 were labelled as high quality, studies that scored ≤ 11/16 labelled as ‘low quality’.

## Results

### Literature search

**Fig. 1:**
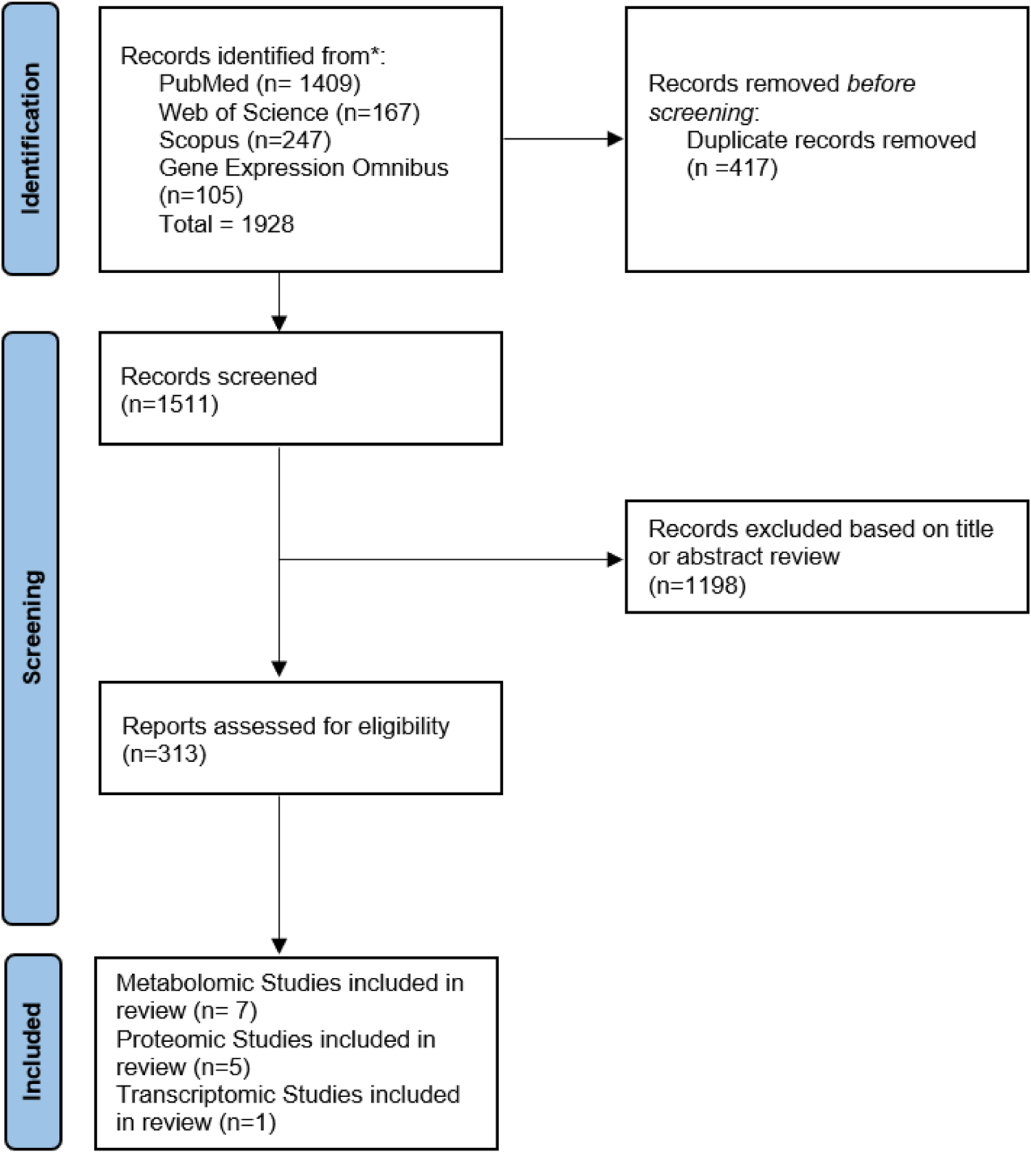
PRISMA diagram PRISMA diagram demonstrating search strategy and final articles included for literature review. After searching, 7 metabolomic papers, 5 proteomic studies and 1 transcriptomic study were included

The study selection process is detailed in the PRISMA chart provided in Fig. 1. Initially, 1,928 studies were identified. After duplicates were removed, 1511 studies remained. Title and abstract screening filtered further studies based on a lack of relevance to the specific research question. Full texts were subsequently interrogated (*n* = 313) and were removed from consideration if there was a lack of suitable data or an irrelevant patient population. Following application of the eligibility criteria, this lead to a total of 13 studies being further investigated.

### Study characteristics

#### Metabolomic studies

Seven studies were identified that have reported the difference in metabolic profile during the course of NAT(Jia et al. 2018; Lv et al. 2022; Rodríguez-Tomàs et al. 2021; Sánchez-Vinces et al. 2023; Strybel et al. 2022; Wang et al. 2022a, b, c; Zhou et al. 2022) (Jia et al. 2018; Lv et al. 2022; Rao et al. 2021; Rodríguez-Tomàs et al. 2021; Strybel et al. 2022; Wang et al. 2022a, b, c; Zhou et al. 2022) and are summarised in Table 1. Most studies (except one (H. Zhou et al. 2022) performed an untargeted analysis, where liquid chromatography-mass spectrometry (LC-MS) was the most utilised analytical platform except for one study(Rodríguez-Tomàs et al. 2021) which used gas chromatography-mass spectrometry (GC-MS). Six of these studies(Jia et al. 2018; Lv et al. 2022; Rodríguez-Tomàs et al. 2021; Sánchez-Vinces et al. 2023; Wang et al. 2022a, b, c; Zhou et al. 2022) included the use of a chemotherapy regime in addition to radiotherapy, where fluoropyrimidine-based treatment was used in all. All studies (apart from two (Sánchez-Vinces et al. 2023; H. Zhou et al. 2022) analysed metabolites in plasma or serum samples (plasma, *n* = 1; serum, *n* = 6) rather than in tumour tissue specimens, emphasizing the relevance of these metabolites in circulation. Dosage of radiotherapy varied minimally across studies (39–50.4 Gy). 29 metabolites were deemed to be statistically different (increased or decreased in abundance) (*p* < 0.05) in the patient response to neoadjuvant therapy in two or more studies. These were taken from the various studies identified from the literature search of which the full list of metabolites can be examined in Supplementary Table 1.

**Table 1:** List of metabolomic studies examining the response of locally advanced rectal cancer detailing chemoradiotherapy regimes, type of sample taken, metabolomic platform analysed on and number of sampling timepoints used

Author	Year	(*n*)	Mean age	Radiotherapy	Chemotherapy	Response Evaluation to Treatment	Platform	Specimen	Targeted/Untargeted	Number of significant metabolites	Number of Sampling Timepoints
Jia	2018	105	-	50 Gy	Capecitabine	TRG	LC-MS	Serum	Untargeted	13	5
Rodriguez-Tomas	2021	32	67.9	50.4 Gy	Capecitabine	Dworak	GC-EI-QTOF-MS	Plasma (EDTA) and Serum	Targeted	21	3
Zhou	2022	14	-	50 Gy	Capecitabine	TRG	UHPLC-MS/MS	Tissue	Untargeted	72	2
Lv	2022	106	51.8	50 Gy	Capecitabine	TRG	UPLC-TOF-MS	Serum	Untargeted	155	5
Wang	2022	165	53.48	50 Gy	Capecitabine + Irinotecan	TRG	LC-MS	Serum	Untargeted	219	5
Strybel	2022	40	65.9	54 Gy	N/A	TRG	GC-MS	Plasma	Untargeted	49	2
Sanchez-Vinces	2023	13	66.8	50.4 Gy	5-FU	TNM staging	LC-MS (reversed phase only)	Tissue	Untargeted	250	2

*UHPLC-MS* Ultra high performance liquid chromatography mass spectrometry,* LC-MS* Liquid chromatography mass spectrometry,* EDTA* Ethylenediaminetetraacetic acid,* GC-EI-QTOF-MS* Gas chromatography electroionisation quadrapole time of flight mass spectrometry,* UPLC-TOF-MS* Ultra performance liquid chromatography time of flight mass spectrometry,* TRG* Tumour regression grade

#### Proteomic studies

Five studies(Bowden et al., 2018; D’Angelo et al., 2023; Rao et al., 2021; Strybel et al., 2022; Wang et al., 2022a, b, c) were identified that utilised proteomics to demonstrate the change in proteomic profile during NAT and are summarised in Table 2. One studiy performing proteomic profiling also performed metabolomic profiling(Strybel et al., 2022) (Rao et al., 2021; Strybel et al., 2022). Another two studies utilised tumour tissue sampling (Bowden et al., 2018; D’Angelo et al., 2023), whilst the remaining three used serum samples. All proteomic studies except for one(Rao et al., 2021) utilised chemotherapy in addition to radiotherapy where most studies used fluoropyrimidine-based chemotherapy. Dosage of radiotherapy varied minimally across studies (45–50 Gy). Patient samples were taken a minimum of two times during NAT in all but three studies (Chauvin et al., 2018; Lee et al., 2022; J. Wang et al., 2022a, b, c). 948 proteins were identified to play a statistically significant role (*p* < 0.05). After removal of duplicates 709 proteins were included in the analysis, the full list can be examined in Supplementary Table 1.

**Table 2:** List of proteomic and transcriptomic studies examining the response of locally advanced rectal cancer detailing chemoradiotherapy regimes, type of sample taken, and number of sampling timepoints used

Author	Year	(*n*)	Mean age	Radiotherapy dose/Gy	Chemotherapy	Response Evaluation to Treatment	Specimen	Number of significant proteins	Number of Sampling Timepoints
D’Angelo	2023	21	61	50.4	5-FU + Oxaliplatin	TRG	Tissue	7	2
Strybel	2022	40	65.9	39 42 54	Unspecified	TRG	Serum	20	2
Bowden	2018	8	74	45-50.4	Capecitabine	TRG	Tissue	27	3
Wang H	2022	13	55	50.6	Capecitabine	TRG	Serum	139	2
Rao	2021	20	51	N/A	N/A	TRG	Serum	526	2
Supiot (Transcriptomic Study)	2013	6	N/A	45	Nil	N/A	Tissue	2	2

*TRG* Tumour regression grade

#### Transcriptomic studies

Only one study (Supiot, 2013) was identified to use transcriptomics (at multiple timepoints) to study the response to NAT and is summarised in Table 2. Supplementary Table 1 details all gene symbols found to be statistically significant (*p* < 0.05) in the prediction of “good” or “poor” responders to NAT.

### Pathway analysis of metabolomic studies

**Fig. 2:**
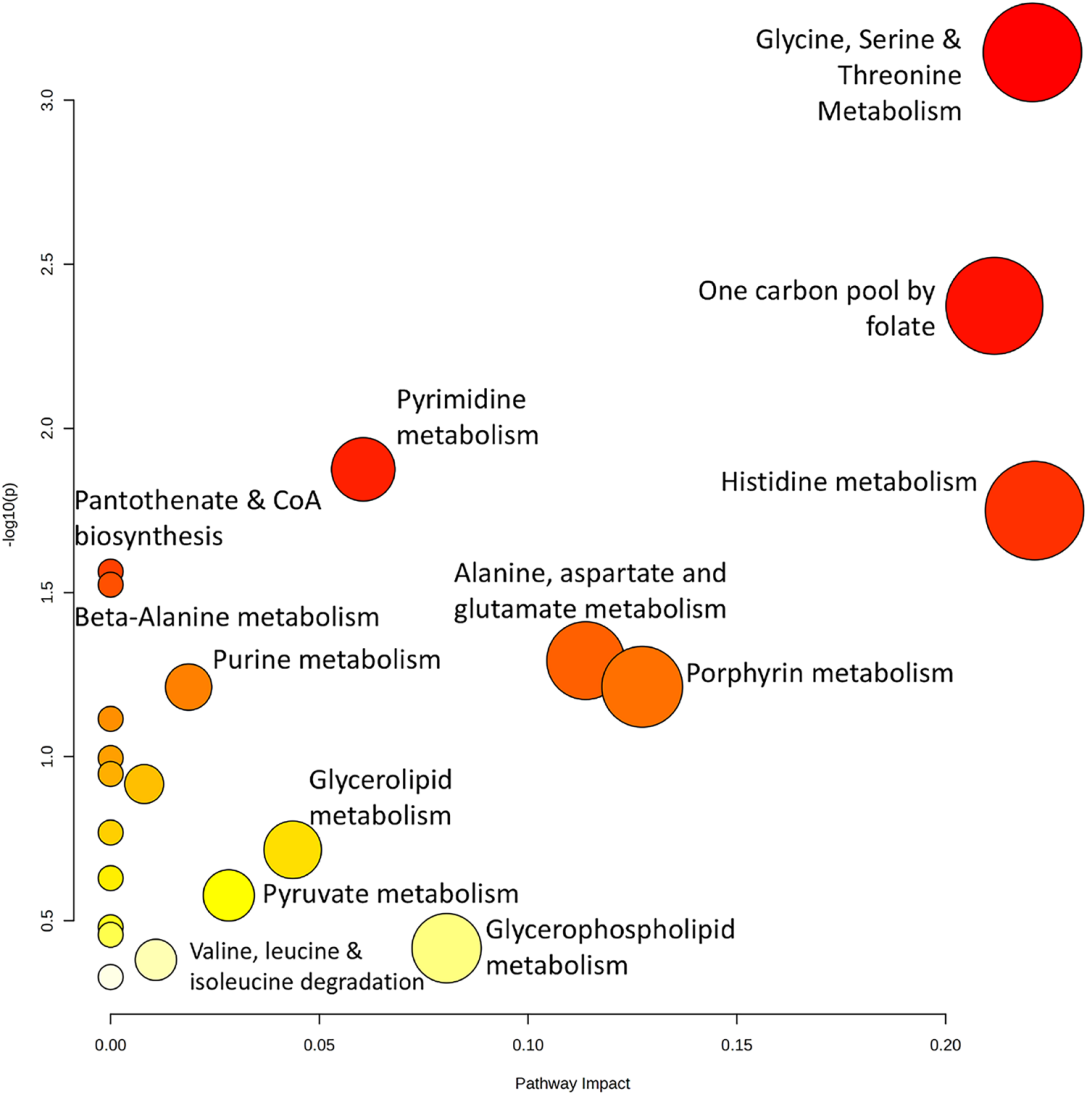
Metabolomic pathway analysis. Scatterplot of metabolic pathway analysis of metabolites in all metabolomic studies (Table 1) created using MetaboAnalyst 6.0. The y-axis depicts the p-value and statistical significance of the pathway, whilst the x-axis depicts the pathway impact illustrating the biological relevance and influence of the pathway. Colour of scatterplot ranging from yellow to red represents the increasing statistical significance and impact of the individual pathway. The most significant pathways include glycine, serine and threonine metabolism, and one carbon pool by folate. The most significant pathways are annotated in the Figure, whilst full data on metabolomic pathway analysis is available in Supplementary Table 2

Following pathway analysis of the identified statistically different 29 metabolites derived from metabolomic studies, seven pathways were shown to have a p-value ≤ 0.05 in relation to the prediction of the neoadjuvant response. Many pathways were amino acid related and included alanine, aspartate and glutamate metabolism, valine, leucine and isoleucine degradation, pyruvate metabolism, glyoxylate metabolism, arginine biosynthesis, glycine, serine and threonine metabolism (the most significant pathway) and glycerophospholipid metabolism. Additionally, pathways related to nucleotide metabolism were present such as one carbon pool by folate, pyrimidine metabolism and purine metabolism. A summary of these pathways is illustrated in Fig. 2, with a full list available in Supplementary Table 2.

### Proteomic pathway analysis

**Fig. 3:**
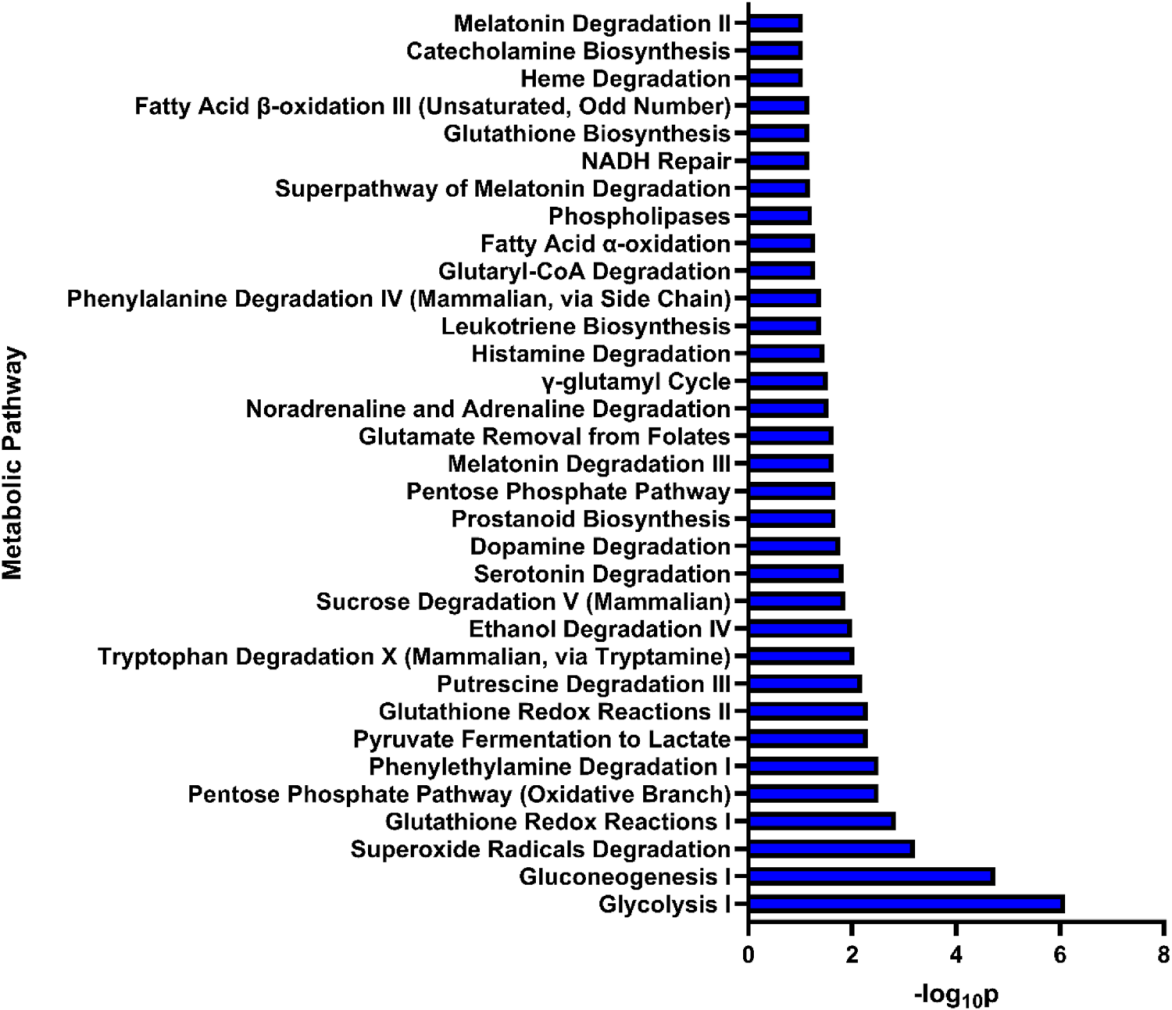
Individual proteomic analysis of metabolic pathway. Bar chart of metabolic pathways using data from proteomic studies demonstrated in Table 2. Pathway analysis performed using QIAGEN Ingenuity Pathway Analysis (IPA). The top 25 pathways are represented on the y-axis, whilst the x-axis represents the p-value and the overall significance of the pathway. The three most significant pathways were glycolysis, gluconeogenesis and superoxide radicals degradation. Full data on proteomic pathway analysis is available in Supplementary Table 3

To validate the findings from the pathway analysis conducted using metabolomics data, a complementary pathway analysis was performed using proteomic data, focusing on the associated metabolite pathways. Individual proteomic pathway analysis is provided in Fig. 3, with full data available in Supplementary Table 3. The three most significant pathways can be demonstrated to be gluconeogenesis, glycolysis, and glutathione-related metabolism in relation to ROS (which are typically generated from radiation treatment).

### Joint pathway analysis

To reinforce the findings from the separate pathway analysis of the metabolome, proteome and transcriptome datasets, a joint pathway analysis (focussed on metabolic pathways only) was performed using the collected list of metabolites as well as a list of genes acquired similarly.

**Fig. 4:**
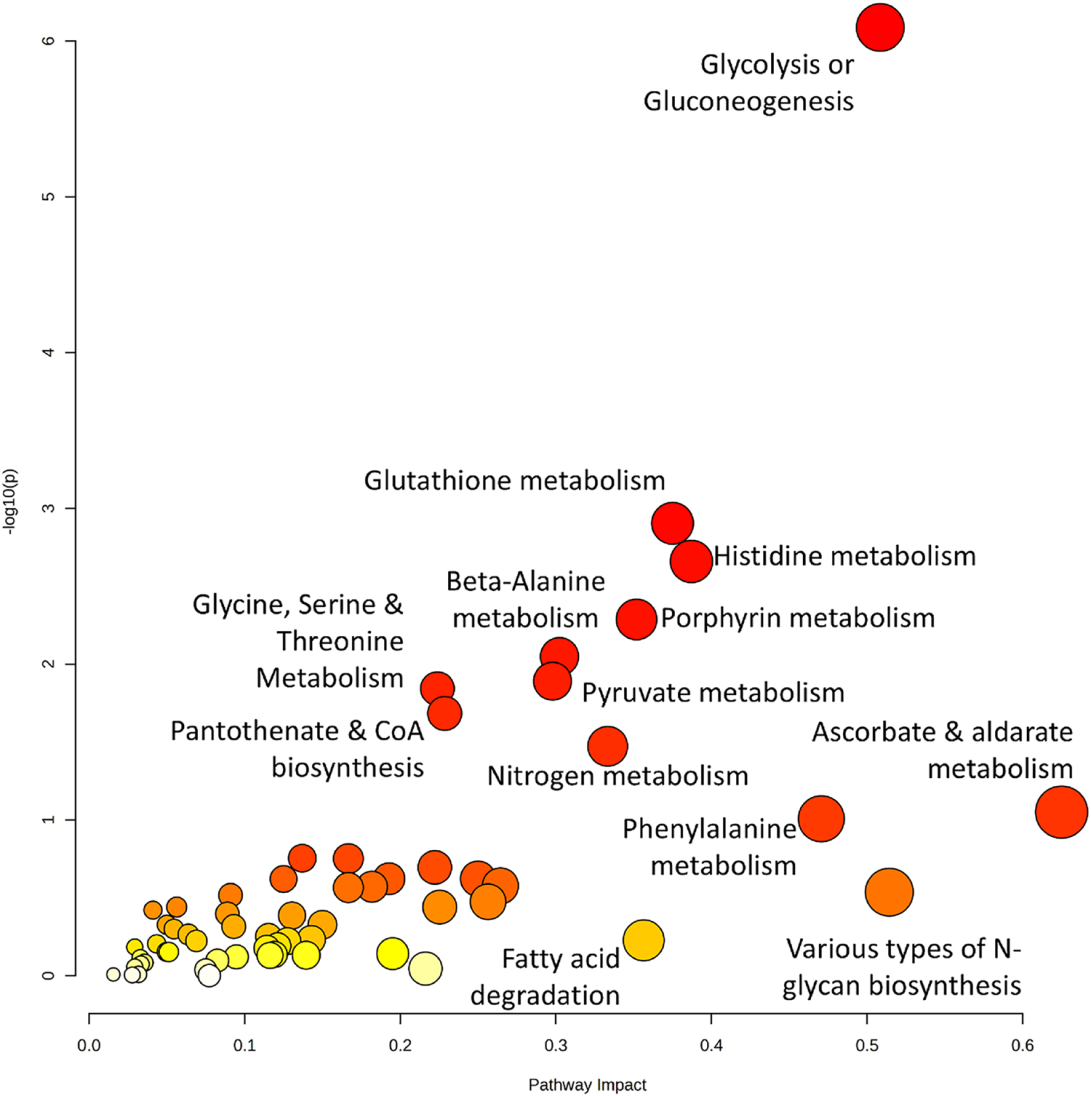
Joint pathway analysis. Scatterplot of joint pathway analysis of metabolic pathways in all metabolomic, proteomic and transcriptomic studies (Tables 1 and 2) created using MetaboAnalyst 6.0. The y-axis depicts the p-value and statistical significance of the pathway, whilst the x-axis depicts the pathway impact illustrating the biological relevance and influence of the pathway. Colour of scatterplot ranging from yellow to red represents the increasing statistical significance and impact of the individual pathway. Full data on joint pathway analysis is available in Supplementary Table 4

All pathways identified in the metabolite-only pathway analysis were also present in the joint pathway analysis, which primarily incorporated proteomic data. The exhaustive list of pathways form the joint pathway analysis is available in Supplementary Table 4. Notable shared pathways included glutathione metabolism, glycine, serine and threonine metabolism, and glycolysis.

### Methodological quality risk of bias

Supplementary Fig. 1 shows the results of the risk of bias assessment using the QUADOMICS tool. Supplementary Table 5 provides a full breakdown of each individually assessed variable.

## Discussion

This review has revealed different metabolic pathways that could influence the response to NAT. The most significant metabolic pathways affected were amino acid metabolism, glutathione-ROS metabolism as well as glycolysis and gluconeogenesis. Dysregulation of these metabolic pathways may contribute to metabolic reprogramming that enables rapid cellular and affords cancer cell survival advantages during treatment. These reprogramming responses could also favour the generation of more NADPH to reduce oxidised glutathione, maintaining high levels of reduced glutathione to support ROS scavenging and mitigate the formation of dsDNA breaks or enhance the generation of fuel and biosynthetic precursors to support proliferation. While these interpretations are biologically plausible and supported by broader cancer literature, it is important to note that the included studies did not typically provide detailed mechanistic explanations. As such, the functional roles discussed below represent hypotheses inferred from established cancer biology rather than conclusions drawn directly from the reviewed data and should be considered as proposals for future experimental validation.

Glycolysis is one of the most significant pathways identified in joint pathway analysis, however, its importance is unsurprising as the Warburg effect is effectively aerobic glycolysis. Additionally, the aerobic glycolysis will fuel auxiliary pathways like the pentose phosphate pathway (Alfarouk et al., 2020) during conversion of glucose to lactate to increase NADPH production.

Proteomic pathway analysis (Fig. 3) and joint pathway analysis (Supplementary Table 4) also reinforced the pentose phosphate pathway’s biological and statistical significance within the response to NAT. Its biological relevance can be defined by generation of ribose and NADPH from glucose-6-phosphate by glucose-6-phosphate dehydrogenase and 6-phosphogluconate by 6-phosphogluconate dehyrogenase. Whilst ribose contributes to nucleotide synthesis (Heiden et al. 2009), the excessive NADPH from this pathway (which accounts for 60% of total NADPH generation (Spaans et al. 2015)) is key for the intracellular redox balance, contributing to maintenance of the pool of reduced glutathione, which will lower ROS levels and lessen the impact of dsDNA breaks. The importance of glutathione metabolism is not only confirmed by proteomic pathway analysis (Fig. 3) but also in joint pathway analysis (Fig. 4). Furthermore, the ribose utilised in nucleotide synthesis provides the building blocks which aids in DNA damage repair caused by dsDNA breaks due to radiation (W. Zhou et al. 2020). Considering the large NADPH pool generated by the pentose phosphate pathway, targeting this process could improve the radiosensitivity of LARC. Inhibition of one of these NADPH-generating enzymes, glucose-6-phosphate dehydrogenase, has been demonstrated in breast cancer in both 2D cellular models as well as a nude mouse model (Mele et al. 2018) to have an excellent inhibitory proliferative effect.

Although aerobic glycolysis can provide survival advantages to LARC during NAT, standard oxidative phosphorylation through the TCA cycle (Fig. 4) will contribute a significant role to the metabolic reprogramming of CRC cells to neutralise ROS. The typical TCA pathway sees acetyl coenzyme (ACoA) enter the cycle after conversion from pyruvate or acetate from fatty acid beta-oxidation and yield NADH and FADH_2_ for use in the electron transport chain. However, the TCA cycle will also simultaneously supply precursors for other biological pathways (Owen et al., 2002), as well as provide more NADPH production through the reductive carboxylation pathway. This begins in the cytoplasm when citrate is converted to 2-ketoglutarate with the assistance of IDH1 in the cytosol (Mycielska et al., 2009). Any excessive citrate can enter the mitochondria and generate NADPH by mitochondrial IDH 2(Rydström, 2006) during another citrate conversion to 2-ketoglutarate. Therefore, the regulation of the TCA cycle can also contribute to metabolic reprogramming as well as produce NADPH for reduction of oxidised glutathione providing a greater survival advantage to radiation exposure. IDH inhibitors have been trialled in AML with higher degrees of success in mutant IDH1 and IDH2(Liu & Gong, 2019) (without cytotoxicity) and thus could represent an intriguing radiosensitising supplement to NCRT.

Serine and glycine were found to be enriched in both metabolic-only and joint pathway analysis (Figs. 2 and 4). Serine plays a central role in supporting anabolic processes critical for tumour growth and survival, due to its links to glycine production and one-carbon metabolism. Serine can be synthesised de novo from the glycolytic intermediate 3-phosphoglycerate (3-PG) via three enzymatic steps, diverting carbon flux from glycolysis (DeBerardinis, 2011). Once formed, serine can be converted into glycine using serine hydroxymethyltransferase (SHMT) (Nonaka et al., 2019) which contributes to the folate cycle, where it donates a one-carbon unit to tetrahydrofolate (THF), generating 5,10-methylene-THF (5,10-mTHF) (Korimerla & Wahl, 2022). This metabolite supports thymidine and pyrimidine biosynthesis and may be further converted into 10-formyl-THF, contributing to de novo purine synthesis (Korimerla & Wahl, 2022). Notably, radiation has been shown to reprogram one-carbon metabolism to favour nucleotide production over methylation, upregulating enzymes such as dihydrofolate reductase (DHFR) and thymidylate synthase (TS) (Batra et al., 2004), while downregulating methylenetetrahydrofolate reductase (MTHFR) to limit methionine cycling and support DNA repair (Batra et al., 2004).

In parallel, one-carbon metabolism contributes to redox homeostasis through the transsulfuration pathway (Zhu et al., 2019). Here, serine-derived homocysteine is converted into cystathionine and subsequently into cysteine, which combines with glutamate to form γ-glutamylcysteine (Aquilano et al., 2014; Zitka et al., 2012). This intermediate is then conjugated with glycine, also derived from serine metabolism, to form glutathione (Aquilano et al., 2014; Zitka et al., 2012). By fuelling both nucleotide synthesis and antioxidant defence, the serine–glycine–cysteine axis enables tumour cells to mitigate radiation-induced oxidative stress. Consistent with this function, glutathione metabolism and glycine, serine, and threonine metabolism were significantly enriched in our pathway analysis, highlighting their potential role in mediating treatment resistance in LARC.

Other metabolic pathways involved in generation of “fuel” for cancer growth include the degradation of branched chain amino acids (BCAA) such as leucine, isoleucine and valine(Fig. 2). BCAAs undergo transamination by branched-chain amino acid transferase (BCAT 1/2) to a branched chain keto acid (BCKA) following nitrogen transfer to alpha-ketoglutarate (forming glutamate) (Sivanand & Vander Heiden, 2020). These BCKAs can then be metabolised into acetyl-CoA and succinyl-CoA, intermediates that enter the TCA cycle, supporting ATP production and biosynthesis in rapidly proliferating cancer cells.(Sivanand & Vander Heiden, 2020).

Importantly, dysregulation of BCAA metabolism has also been implicated in oncogenic signalling. Leucine, for example, is a known activator of the mammalian target of rapamycin complex (mTORC), a central regulator of cell growth and proliferation (Ananieva et al., 2016). In chronic myeloid leukaemia (CML), increased BCAT1 expression has been shown to elevate intracellular BCAA levels and activate the mTORC pathway) (Zhang & Han, 2017; Zheng et al., 2016). Similar findings have been reported in breast cancer, where BCAT1 expression correlates with increased BCAA levels in tumour tissue and serum, and mTORC activity (Hattori et al., 2017).(L. Zhang & Han, 2017). Targeting BCAT 1 through BCAT 1 inhibitors has already been demonstrated an increased sensitivity to cisplatin in hepatocellular and cervical cancer cells (Luo et al., 2021). Together, these findings suggest that enhanced BCAA degradation, via upregulated BCAT activity and downstream catabolic flux, may represent a key feature of metabolic reprogramming in poor responders to neoadjuvant therapy in LARC. This metabolic axis may fuel tumour growth while also supporting pro-survival signalling through mTORC activation (Kang et al., 2024; Najumudeen et al., 2021).

Alanine, aspartate and glutamate (AAG) metabolism has been identified as a crucial player in the response to NAT as shown in pathway and joint pathway analysis (Figs. 2 and 4). However, alanine’s role in cancer cell proliferation and survival is only limited to pyruvate synthesis, through L-alanine catabolism via the enzyme alanine aminotransferase 2 (ALT 2) (Hodakoski et al., 2019). Despite this solitary function, pyruvate generation is an important element of to satisfy the proliferative needs of cancer cells and as such ALT 2 has been inhibited to limit this pyruvate generation with great effect in nude mouse metastatic breast cancer models (Elia et al., 2019), making this enzyme target highly intriguing for neoadjuvant-treated LARC patients.

Aspartate metabolism, on the other hand, plays a much larger role in sustaining rapid proliferation of these colorectal cells. This occurs through the increased production of DNA and replication by upregulating purine and pyrimidine nucleotide (Coloff et al. 2016) synthesis, as well as providing the building blocks for DNA repair due to radiation-induced dsDNA breaks.The glutamate generated from BCAA transamination and the AAG metabolic pathway can also improve survival from radiation exposure as well as being independently involved in the NAT response (Figs. 2 and 4). Glutamate can be converted to asparagine (Huang et al. 2017), where asparagine has been demonstrated to suppress apoptosis (J. Zhang et al. 2014). Typically, when cancer cells are exposed to ionising radiation, dsDNA breaks can activate the p53 pathway and begin the transcription of apoptotic genes (Jiao et al. 2022). Asparagine-induced apoptotic suppression, therefore, can represent a potential radioresistant mechanism for NAT by preventing cell radiation-induced cell death. Glutamate metabolism will also contribute to protection against radiation-induced DSBs through NADPH generation using two mechanisms: the reductive carboxylation (Jiang et al. 2016; Son et al. 2013) pathway (described above) and a noncanonical pathway that generates NADPH through glutaminolysis through the mitochondrial glutamine transporter. This will assist the reduction of oxidised glutathione to reduced glutathione to reduce concentrations of harmful ROS (Son et al. 2013). Proteomic pathway analysis has also supported this, as superoxide radical degradation was the third most significant pathway (Fig. 3), illustrating that increased antioxidation of ROS to be typical of radioresistant tumours. Preventing glutamate uptake therefore could represent a strategy to stem this dependence on glutamate.

Finally, as glycerophospholipid metabolism is important during NAT (Figs. 2 and 4), it is plausible that cell membrane generation, of which glycerophospholipids are a key component (Hishikawa et al., 2014), is pivotal for survival and proliferation of rectal cancer cells. As glycerophospholipid metabolism contributes to cell membrane formation, its relevance to rapidly proliferating cancer cells is a promising avenue for further investigation. Glycerophospholipid levels have also been shown to link to the progression of CRC in a mouse model (Francipane & Lagasse, 2014; Tian et al., 2019), representing the importance of glycerophospholipid metabolism in proliferation. Furthermore, ROS can induce lipid peroxidation (Su et al., 2019), further emphasising the importance of glycerophospholipid metabolism.

In recent years, there has been a trend toward a non-operative approach in patients that have a clinical complete response to total neoadjuvant therapy (TNT), however, the implications of TNT on the identified important metabolic pathways in this review are still unknown. No studies fulfilling the inclusion criteria of this review have examined the use of a TNT regime on patient metabolism. Only three studies (Jia et al., 2018; Lv et al., 2022; Wang et al., 2022a, b, c) have attempted to identify discriminatory metabolic biomarkers that elucidate a pCR, but these studies have all employed a traditional long course chemoradiotherapy regime. Furthermore, there is no consistency in these predictive biomarkers between studies. Additionally, no study is yet to demonstrate how any metabolic biomarkers relate to core clinical outcomes (except for pCR) such as operative morbidity or mortality as well as overall survival (OS). Therefore, a knowledge gap is present in the literature in correlating metabolomic profiles with core clinical outcomes.

Although this review has identified key metabolic pathways activated in response to the stress of NAT, there are also limitations. Firstly, the metabolite profiles of patients responding differently to NAT was significantly varied. For example, Wang et al. found modified levels of multiple essential and BCAAs, which can be explained by the detection bias of different analytical platforms and assays used to carry out each respective study’s analysis (Table 1). Despite this, lipid metabolism appears heavily implicated in the response to neoadjuvant therapy as a common theme amongst most of the included studies (Supplementary Table 2). There was also a variety in clinical populations and treatment. Tables 1 and 2 demonstrates the profiles of the studies included and shows differences in the platform used, chemotherapeutic regimes as well as a variety in the number of participants recruited. As a result, the number of statistically significant biomarkers is also varied considerably between studies. Furthermore, the sample types varied between studies. Table 1 shows that most metabolomic studies exclusively used a blood-based sample type whereas the proteomic and transcriptomic studies (Table 2) used a mix of blood-based and tumour samples. This could be responsible for the differences in reported statistically different metabolites depending on sample type. For example, a positively statistically different metabolite in the serum may be negatively statistically different in the tissue specimen.

The number of timepoints for sample collection varied between studies. Whilst metabolomic and proteomic studies had a majority of at least two timepoints for sample collection, all transcriptomic studies only collected samples prior to patients starting NAT without any further timepoints. Lastly, the studies included in this review did not all disclose their fold change values for all metabolites, proteins and genes. This did not allow any quantitative analysis or pathway enrichment analysis and therefore, this is a qualitative systematic review rather than a quantitative meta-analysis. Therefore, identifying specific metabolite biomarkers to pursue further research from this data cannot be qualified, thus making the conclusions of this article highly preliminary.

## Conclusion

In conclusion, this review has identified which metabolic pathways are altered in the patient response to NAT in LARC, given the available data in the literature. These pathways can reprogramme to support tumour proliferation by increasing cellular fuel availability, neutralising toxic ROS to reduce dsDNA breaks, and enhancing biosynthesis of essential organelle components. These metabolic adaptations may enable cancer cell survival and growth under treatment-induced stress, reflecting core mechanisms of radioresistance.

Importantly, our conclusions are limited by two major constraints: the scarcity of eligible transcriptomic data, which restricted full multi-omic integration; and the narrow focus of included studies on short-term treatment outcomes, such as pCR and TRG. None of the reviewed studies examined relationships between metabolic changes and longer-term clinical endpoints like overall survival, disease-free survival, or local recurrence. This limits the immediate clinical translatability and prognostic utility of identified biomarkers.

Future work is therefore needed not only to validate these metabolic pathways experimentally, for example, by profiling cancer cell metabolomes following ionising radiation, but also to incorporate longitudinal clinical follow-up. Bridging this gap will be essential for the development of metabolism-informed therapeutic strategies that offer real, long-term benefit to patients with LARC.

## Supplementary Information

Below is the link to the electronic supplementary material.

Supplementary Material 1

Supplementary Material 2

Supplementary Material 3

Supplementary Material 4

Supplementary Material 5

Supplementary Material 6

## Data Availability

No datasets were generated or analysed during the current study.
