# Supplementary material for: A systematic review of omics discovery studies to identify pertinent metabolic pathways for locally advanced rectal cancer in response to neoadjuvant chemoradiotherapy
Source: Metabolomics. 2025 Aug 21;21(5):124. doi: 10.1007/s11306-025-02319-y (PMC12370562; doi:10.1007/s11306-025-02319-y)
Supplement: Supplementary file 6 — Supplementary Material 6 [file 11306_2025_2319_MOESM6_ESM.docx]

**Supplementary Figure 1:** Risk of bias assessment using QUADOMICS evaluation of the quality of studies included in the systematic review. QUADOMICS criteria defined in Supplementary Table 5.

| **Author** | **Year** | **Item** | | | | | | | | | | | | | | | |
| --- | --- | --- | --- | --- | --- | --- | --- | --- | --- | --- | --- | --- | --- | --- | --- | --- | --- |
|  |  | **1** | **2** | **3** | **4** | **5** | **6** | **7** | **8** | **9** | **10** | **11** | **12** | **13** | **14** | **15** | **16** |
| Jia | 2018 |  |  |  |  |  |  |  |  |  |  |  |  |  |  |  |  |
| Rodriguez-Tomas | 2021 |  |  |  |  |  |  |  |  |  |  |  |  |  |  |  |  |
| Zhou | 2022 |  |  |  |  |  |  |  |  |  |  |  |  |  |  |  |  |
| Lv | 2022 |  |  |  |  |  |  |  |  |  |  |  |  |  |  |  |  |
| Wang | 2022 |  |  |  |  |  |  |  |  |  |  |  |  |  |  |  |  |
| Rao | 2021 |  |  |  |  |  |  |  |  |  |  |  |  |  |  |  |  |
| Strybel | 2022 |  |  |  |  |  |  |  |  |  |  |  |  |  |  |  |  |
| D’Angelo | 2023 |  |  |  |  |  |  |  |  |  |  |  |  |  |  |  |  |
| Bowden | 2018 |  |  |  |  |  |  |  |  |  |  |  |  |  |  |  |  |
| Wang H | 2022 |  |  |  |  |  |  |  |  |  |  |  |  |  |  |  |  |
| Ghadimi | 2005 |  |  |  |  |  |  |  |  |  |  |  |  |  |  |  |  |
| Rimkus | 2008 |  |  |  |  |  |  |  |  |  |  |  |  |  |  |  |  |
| Supiot | 2013 |  |  |  |  |  |  |  |  |  |  |  |  |  |  |  |  |
| Sanchez-Vinces | 2023 |  |  |  |  |  |  |  |  |  |  |  |  |  |  |  |  |

|  | **Yes** |
| --- | --- |
|  | **Unsure** |
|  | **No** |
